# *catena*-Poly[[tetra­aqua­cadmium]-μ-5,5′-(1,4-phenyl­ene)di(tetra­zol-2-ido)-κ^2^ *N* ^2^:*N* ^2′^]

**DOI:** 10.1107/S1600536813010441

**Published:** 2013-04-20

**Authors:** Qinqin Dang, Han Caiyun

**Affiliations:** a School of Chemistry & Material Science, Shanxi Normal University, Linfen 041004, People’s Republic of China

## Abstract

In the title compound, [Cd(C_8_H_4_N_8_)(H_2_O)_4_]_*n*_, 5,5′-(1,4-phenyl­ene)di(tetra­zol-2-ide) (*L*) ligands bridge Cd^II^ atoms into polymeric chains along [201]. The Cd^II^ atom is situated on an inversion centre and is coordinated by two N atoms from two *L* ligands and by four water O atoms in a distorted octa­hedral geometry. In the *L* ligand, the benzene ring resides on an inversion centre and the tetra­zole rings are twisted from its plane by 22.3 (1)°. An extensive hydrogen-bonding network formed by classical O—H⋯N and O—H⋯O inter­actions consolidates the crystal packing, linking the poymeric chains into a three-dimensional structure.

## Related literature

For background to coordination frameworks, see: Yaghi *et al.* (2003 ▶); Kitagawa *et al.* (2004 ▶); Ockwig *et al.* (2005 ▶). For details of the synthesis of 1,4-bis­(tetra­zole-5-yl)benzene, see: Tao *et al.* (2004 ▶). For the crystal structures of coordination polymers containing the 1,4-bis­(tetra­zole-5-yl)benzene ligand, see: Dinca *et al.* (2006 ▶); Ouellette *et al.* (2009 ▶); Liu *et al.* (2012 ▶).
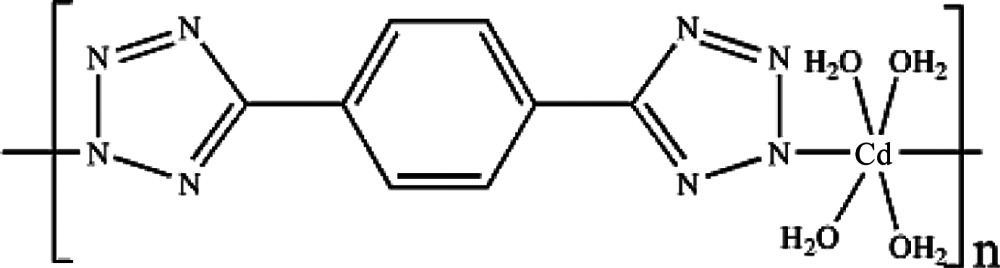


## Experimental

### 

#### Crystal data

[Cd(C_8_H_4_N_8_)(H_2_O)_4_]
*M* *_r_* = 396.66
Monoclinic, 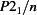

*a* = 5.3188 (4) Å
*b* = 11.1525 (14) Å
*c* = 12.0279 (8) Å
β = 101.256 (7)°
*V* = 699.75 (11) Å^3^
*Z* = 2
Mo *K*α radiation
μ = 1.59 mm^−1^
*T* = 293 K
0.25 × 0.20 × 0.15 mm

#### Data collection

Agilent Xcalibur (Eos, Gemini) diffractometer
Absorption correction: multi-scan (*CrysAlis PRO*; Agilent, 2012 ▶) *T*_min_ = 0.692, *T*_max_ = 0.796
2351 measured reflections
1237 independent reflections
895 reflections with *I* > 2σ(*I*)
*R*_int_ = 0.033

#### Refinement

*R*[*F* ^2^ > 2σ(*F* ^2^)] = 0.040
*wR*(*F* ^2^) = 0.085
*S* = 1.05
1237 reflections
99 parameters
H-atom parameters constrained
Δρ_max_ = 0.62 e Å^−3^
Δρ_min_ = −0.53 e Å^−3^

### 

Data collection: *CrysAlis PRO* (Agilent, 2012 ▶); cell refinement: *CrysAlis PRO*; data reduction: *CrysAlis PRO*; program(s) used to solve structure: *SHELXS97* (Sheldrick, 2008 ▶); program(s) used to refine structure: *SHELXL97* (Sheldrick, 2008 ▶); molecular graphics: *DIAMOND* (Brandenburg & Putz, 2006 ▶); software used to prepare material for publication: *SHELXTL* (Sheldrick, 2008 ▶).

## Supplementary Material

Crystal structure: contains datablock(s) I, global. DOI: 10.1107/S1600536813010441/cv5403sup1.cif

Structure factors: contains datablock(s) I. DOI: 10.1107/S1600536813010441/cv5403Isup2.hkl

Additional supplementary materials:  crystallographic information; 3D view; checkCIF report

## Figures and Tables

**Table 1:** Hydrogen-bond geometry (Å, °)

*D*—H⋯*A*	*D*—H	H⋯*A*	*D*⋯*A*	*D*—H⋯*A*
O1—H1*B*⋯N1^i^	0.86	1.93	2.779 (5)	167
O1—H1*A*⋯N4^ii^	0.86	1.99	2.836 (6)	166
O2—H2*A*⋯N3^iii^	0.85	2.49	3.121 (6)	131
O2—H2*B*⋯O1^iv^	0.85	2.39	3.035 (6)	133

Symmetry codes: (i) 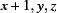; (ii) 
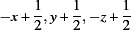
; (iii) 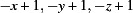; (iv) 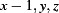.

**Fig. 1.**
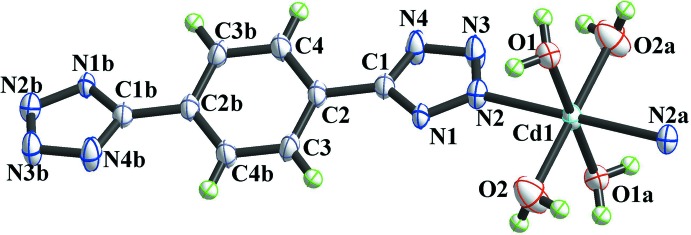
A portion of the polymeric chain in the title compound showing the atomic numbering [symmetry codes: (a) 1-x , 1-y, 1-z; (b) -1-x, 1-y, -z]. Displacement ellipsoids are drawn at the 50% probability level.

## supplementary crystallographic information

### Comment

Over the last decade coordination frameworks with channels or pores have captivated great attention of chemists because of their potential applications in gas storage, separation, ion exchange and catalysis(Yaghi *et al.*, 2003; Kitagawa *et al.*, 2004; Ockwig *et al.*, 2005). 1,4-Bis(tetrazol-5-yl)benzene, firstly synthesized and characterized by Tao *et al.* (2004), is now widely used for constructing coordination frameworks with channels or pores (Dinca *et al.*, 2006; Ouellette *et al.*, 2009; Liu *et al.*, 2012). This paper concerns the reaction of cadmium(II) and 1,4-bis(tetrazol-5-yl)benzene, and the crystal structure of the product.

In the title compound (Fig. 1), the Cd^II^ ion is located at an inversion centre. It has a slightly distorted octahedral coordination geometry formed by four water molecules and two nitrogen atoms from ligands *L*, where H_2_*L* = 1,4-bis(tetrazol-5-yl)benzene. Four oxygen atoms form a planar parallelogram arrangement around the Cd centre, and the other two nitrogen atoms occupy the apical position. Each ligand *L* coordinates two cadmium atoms in a µ_2_-bridging mode, thus generating a one-dimension coordination polymer. As far as we known, this coordination mode is currently unknown for *L* ligand.

In the crystal, polymeric one-dimensional chains are linked *via* O—H···N hydrogen bonds (Table 1) into a three-dimensional structure. The results show that there are no channels in the crystal structure.

### Experimental

Cadmium nitrate tetrahydrate (0.123 g, 0.40 mmol), 1,4-bis(tetrazole-5-yl)benzene (0.042 g, 0.20 mmol) and sodium hydroxide (0.016, 0.40 mmol) were added to 8 ml of water:ammonium hydroxide (v:v=1:1) mixture. The solution was transferred into a Teflon-lined stainless steel autoclave and the autoclave was heated to 393 K and maintained at that temperature for 72 h. After cooling to room temperature, crystals suitable for X-ray diffraction were collected.

### Refinement

Water hydrogen atoms were placed in calculated positions [O—H = 0.85–0.87 Å], and refined as riding, with U_iso_(H) = 1.5 U_eq_(O). The aromatic H atoms were positioned geometrically [C—H = 0.93 Å], and refined using a riding model, with U_iso_(H) = 1.2 U_eq_(C).

### Crystal data

**Table:**

[Cd(C_8_H_4_N_8_)(H_2_O)_4_]	*F*(000) = 392
*M**_r_* = 396.66	2013-04-07 # Formatted by publCIF
Monoclinic, *P*2_1_/*n*	*D*_x_ = 1.883 Mg m^−^^3^
Hall symbol: -P 2yn	Mo *K*α radiation, λ = 0.71073 Å
*a* = 5.3188 (4) Å	Cell parameters from 739 reflections
*b* = 11.1525 (14) Å	θ = 3.5–29.1°
*c* = 12.0279 (8) Å	µ = 1.59 mm^−^^1^
β = 101.256 (7)°	*T* = 293 K
*V* = 699.75 (11) Å^3^	Prism, yellow
*Z* = 2	0.25 × 0.20 × 0.15 mm

### Data collection

**Table:**

Agilent Xcalibur (Eos, Gemini) diffractometer	1237 independent reflections
Radiation source: fine-focus sealed tube	895 reflections with *I* > 2σ(*I*)
Graphite monochromator	*R*_int_ = 0.033
Detector resolution: 16.0710 pixels mm^-1^	θ_max_ = 25.0°, θ_min_ = 3.5°
ω scans	*h* = −5→6
Absorption correction: multi-scan (*CrysAlis PRO*; Agilent, 2012)	*k* = −5→13
*T*_min_ = 0.692, *T*_max_ = 0.796	*l* = −13→14
2351 measured reflections	

### Refinement

**Table:**

Refinement on *F*^2^	Primary atom site location: structure-invariant direct methods
Least-squares matrix: full	Secondary atom site location: difference Fourier map
*R*[*F*^2^ > 2σ(*F*^2^)] = 0.040	Hydrogen site location: inferred from neighbouring sites
*wR*(*F*^2^) = 0.085	H-atom parameters constrained
*S* = 1.05	*w* = 1/[σ^2^(*F*_o_^2^) + (0.0301*P*)^2^] where *P* = (*F*_o_^2^ + 2*F*_c_^2^)/3
1237 reflections	(Δ/σ)_max_ < 0.001
99 parameters	Δρ_max_ = 0.62 e Å^−^^3^
0 restraints	Δρ_min_ = −0.53 e Å^−^^3^

### Special details

**Table:**

Geometry. All e.s.d.'s (except the e.s.d. in the dihedral angle between two l.s. planes) are estimated using the full covariance matrix. The cell e.s.d.'s are taken into account individually in the estimation of e.s.d.'s in distances, angles and torsion angles; correlations between e.s.d.'s in cell parameters are only used when they are defined by crystal symmetry. An approximate (isotropic) treatment of cell e.s.d.'s is used for estimating e.s.d.'s involving l.s. planes.
Refinement. Refinement of *F*^2^ against ALL reflections. The weighted *R*-factor *wR* and goodness of fit *S* are based on *F*^2^, conventional *R*-factors *R* are based on *F*, with *F* set to zero for negative *F*^2^. The threshold expression of *F*^2^ > σ(*F*^2^) is used only for calculating *R*-factors(gt) *etc*. and is not relevant to the choice of reflections for refinement. *R*-factors based on *F*^2^ are statistically about twice as large as those based on *F*, and *R*- factors based on ALL data will be even larger.

### Fractional atomic coordinates and isotropic or equivalent isotropic displacement parameters (Å^2^)

**Table:**

	*x*	*y*	*z*	*U*_iso_*/*U*_eq_	
Cd1	0.5000	0.5000	0.5000	0.0273 (2)	
O1	0.7271 (6)	0.6312 (4)	0.4040 (3)	0.0365 (10)	
H1A	0.6261	0.6859	0.3696	0.055*	
H1B	0.7918	0.5921	0.3544	0.055*	
N1	0.0067 (7)	0.5049 (4)	0.2701 (3)	0.0266 (10)	
N4	0.0598 (8)	0.3375 (4)	0.1802 (4)	0.0369 (12)	
C2	−0.2949 (9)	0.4694 (5)	0.0882 (4)	0.0279 (13)	
C4	−0.3166 (9)	0.4199 (5)	−0.0197 (4)	0.0333 (13)	
H4	−0.1933	0.3658	−0.0335	0.040*	
N2	0.2066 (7)	0.4426 (4)	0.3287 (4)	0.0327 (11)	
C1	−0.0782 (9)	0.4371 (5)	0.1798 (4)	0.0286 (12)	
N3	0.2401 (8)	0.3426 (4)	0.2768 (4)	0.0403 (12)	
O2	0.2732 (8)	0.6693 (4)	0.5307 (4)	0.0659 (14)	
H2A	0.3484	0.7038	0.5914	0.099*	
H2B	0.1225	0.6488	0.5376	0.099*	
C3	−0.4828 (10)	0.5505 (5)	0.1061 (4)	0.0342 (13)	
H3	−0.4724	0.5849	0.1773	0.041*	

### Atomic displacement parameters (Å^2^)

**Table:**

	*U*^11^	*U*^22^	*U*^33^	*U*^12^	*U*^13^	*U*^23^
Cd1	0.0262 (3)	0.0279 (3)	0.0244 (3)	0.0008 (3)	−0.0036 (2)	−0.0009 (3)
O1	0.034 (2)	0.041 (3)	0.034 (2)	0.0078 (17)	0.0051 (18)	0.0042 (19)
N1	0.021 (2)	0.036 (3)	0.019 (2)	0.005 (2)	−0.0059 (17)	0.001 (2)
N4	0.039 (3)	0.033 (3)	0.031 (3)	0.008 (2)	−0.013 (2)	−0.005 (2)
C2	0.023 (3)	0.034 (4)	0.024 (3)	−0.003 (2)	−0.002 (2)	0.001 (2)
C4	0.030 (3)	0.036 (4)	0.030 (3)	0.008 (3)	−0.004 (2)	−0.002 (3)
N2	0.031 (2)	0.039 (3)	0.025 (2)	0.001 (2)	−0.004 (2)	0.001 (2)
C1	0.027 (3)	0.035 (3)	0.020 (3)	0.000 (3)	−0.002 (2)	0.002 (3)
N3	0.042 (3)	0.036 (3)	0.034 (3)	0.005 (2)	−0.014 (2)	−0.003 (2)
O2	0.047 (2)	0.062 (3)	0.084 (4)	0.001 (2)	0.001 (2)	−0.019 (3)
C3	0.039 (3)	0.041 (4)	0.018 (3)	0.006 (3)	−0.006 (2)	−0.006 (3)

### Geometric parameters (Å, º)

**Table:**

Cd1—O2^i^	2.309 (4)	N4—N3	1.355 (5)
Cd1—O2	2.309 (4)	C2—C4	1.394 (7)
Cd1—O1	2.340 (3)	C2—C3	1.396 (7)
Cd1—O1^i^	2.340 (3)	C2—C1	1.475 (7)
Cd1—N2	2.416 (4)	C4—C3^ii^	1.377 (7)
Cd1—N2^i^	2.416 (4)	C4—H4	0.9300
O1—H1A	0.8631	N2—N3	1.307 (6)
O1—H1B	0.8625	O2—H2A	0.8527
N1—C1	1.328 (6)	O2—H2B	0.8526
N1—N2	1.348 (6)	C3—C4^ii^	1.377 (7)
N4—C1	1.331 (6)	C3—H3	0.9300
			
O2^i^—Cd1—O2	179.999 (1)	C4—C2—C3	117.9 (5)
O2^i^—Cd1—O1	95.51 (15)	C4—C2—C1	120.6 (4)
O2—Cd1—O1	84.49 (15)	C3—C2—C1	121.4 (5)
O2^i^—Cd1—O1^i^	84.49 (15)	C3^ii^—C4—C2	121.3 (5)
O2—Cd1—O1^i^	95.51 (15)	C3^ii^—C4—H4	119.4
O1—Cd1—O1^i^	180.0	C2—C4—H4	119.4
O2^i^—Cd1—N2	85.26 (16)	N3—N2—N1	110.9 (4)
O2—Cd1—N2	94.74 (16)	N3—N2—Cd1	120.5 (3)
O1—Cd1—N2	93.12 (13)	N1—N2—Cd1	128.4 (3)
O1^i^—Cd1—N2	86.88 (13)	N1—C1—N4	111.9 (4)
O2^i^—Cd1—N2^i^	94.74 (16)	N1—C1—C2	124.3 (5)
O2—Cd1—N2^i^	85.26 (16)	N4—C1—C2	123.8 (5)
O1—Cd1—N2^i^	86.88 (13)	N2—N3—N4	107.8 (4)
O1^i^—Cd1—N2^i^	93.12 (13)	Cd1—O2—H2A	109.5
N2—Cd1—N2^i^	180.0 (3)	Cd1—O2—H2B	109.1
Cd1—O1—H1A	110.2	H2A—O2—H2B	109.3
Cd1—O1—H1B	109.8	C4^ii^—C3—C2	120.8 (5)
H1A—O1—H1B	108.7	C4^ii^—C3—H3	119.6
C1—N1—N2	104.0 (4)	C2—C3—H3	119.6
C1—N4—N3	105.4 (4)		

Symmetry codes: (i) −*x*+1, −*y*+1, −*z*+1; (ii) −*x*−1, −*y*+1, −*z*.

### Hydrogen-bond geometry (Å, º)

**Table:**

*D*—H···*A*	*D*—H	H···*A*	*D*···*A*	*D*—H···*A*
O1—H1*B*···N1^iii^	0.86	1.93	2.779 (5)	167
O1—H1*A*···N4^iv^	0.86	1.99	2.836 (6)	166
O2—H2*A*···N3^i^	0.85	2.49	3.121 (6)	131
O2—H2*B*···O1^v^	0.85	2.39	3.035 (6)	133

Symmetry codes: (i) −*x*+1, −*y*+1, −*z*+1; (iii) *x*+1, *y*, *z*; (iv) −*x*+1/2, *y*+1/2, −*z*+1/2; (v) *x*−1, *y*, *z*.
